# Effects of metal nanoparticles on tight junction-associated proteins via HIF-1α/miR-29b/MMPs pathway in human epidermal keratinocytes

**DOI:** 10.1186/s12989-021-00405-2

**Published:** 2021-03-19

**Authors:** Jiali Yuan, Yue Zhang, Yuanbao Zhang, Yiqun Mo, Qunwei Zhang

**Affiliations:** Department of Environmental and Occupational Health Sciences, School of Public Health and Information Sciences, University of Louisville, 485 E. Gray Street, Louisville, KY 40202 USA

**Keywords:** Metal nanoparticles, Immortalized human keratinocytes HaCaT, Hypoxia inducible factor-1α (HIF-1α), miR-29b, Matrix metalloproteinases, Tight junction-associated proteins

## Abstract

**Background:**

The increasing use of metal nanoparticles in industry and biomedicine raises the risk for unintentional exposure. The ability of metal nanoparticles to penetrate the skin ranges from stopping at the stratum corneum to passing below the dermis and entering the systemic circulation. Despite the potential health risks associated with skin exposure to metal nanoparticles, the mechanisms underlying the toxicity of metal nanoparticles on skin keratinocytes remain unclear. In this study, we proposed that exposure of human epidermal keratinocytes (HaCaT) to metal nanoparticles, such as nickel nanoparticles, dysregulates tight-junction associated proteins by interacting with the HIF-1α/miR-29b/MMPs axis.

**Methods:**

We performed dose-response and time-response studies in HaCaT cells to observe the effects of Nano-Ni or Nano-TiO_2_ on the expression and activity of MMP-2 and MMP-9, and on the expression of tight junction-associated proteins, TIMP-1, TIMP-2, miR-29b, and HIF-1α. In the dose-response studies, cells were exposed to 0, 10, or 20 μg/mL of Nano-Ni or Nano-TiO_2_ for 24 h. In the time-response studies, cells were exposed to 20 μg/mL of Nano-Ni for 12, 24, 48, or 72 h. After treatment, cells were collected to either assess the expression of mRNAs and miR-29b by real-time PCR or to determine the expression of tight junction-associated proteins and HIF-1α nuclear accumulation by Western blot and/or immunofluorescent staining; the conditioned media were collected to evaluate the MMP-2 and MMP-9 activities by gelatin zymography assay. To further investigate the mechanisms underlying Nano-Ni-induced dysregulation of tight junction-associated proteins, we employed a HIF-1α inhibitor, CAY10585, to perturb HIF-1α accumulation in one experiment, and transfected a miR-29b-3p mimic into the HaCaT cells before Nano-Ni exposure in another experiment. Cells and conditioned media were collected, and the expression and activities of MMPs and the expression of tight junction-associated proteins were determined as described above.

**Results:**

Exposure of HaCaT cells to Nano-Ni resulted in a dose-dependent increase in the expression of MMP-2, MMP-9, TIMP-1, and TIMP-2 and the activities of MMP-2 and MMP-9. However, exposure of cells to Nano-TiO_2_ did not cause these effects. Nano-Ni caused a dose-dependent decrease in the expression of miR-29b and tight junction-associated proteins, such as ZO-1, occludin, and claudin-1, while Nano-TiO_2_ did not. Nano-Ni also caused a dose-dependent increase in HIF-1α nuclear accumulation. The time-response studies showed that Nano-Ni caused significantly increased expressions of MMP-2 at 24 h, MMP-9 at 12, 24, and 48 h, TIMP-1 from 24 to 72 h, and TIMP-2 from 12 to 72 h post-exposure. The expression of miR-29b and tight junction-associated proteins such as ZO-1, occludin, and claudin-1 decreased as early as 12 h post-exposure, and their levels declined gradually over time. Pretreatment of cells with a HIF-1α inhibitor, CAY10585, abolished Nano-Ni-induced miR-29b down-regulation and MMP-2/9 up-regulation. Introduction of a miR-29b-3p mimic into HaCaT cells by transfection before Nano-Ni exposure ameliorated Nano-Ni-induced increased expression and activity of MMP-2 and MMP-9 and restored Nano-Ni-induced down-regulation of tight junction-associated proteins.

**Conclusion:**

Our study herein demonstrated that exposure of human epidermal keratinocytes to Nano-Ni caused increased HIF-1α nuclear accumulation and increased transcription and activity of MMP-2 and MMP-9 and down-regulation of miR-29b and tight junction-associated proteins. Nano-Ni-induced miR-29b down-regulation was through Nano-Ni-induced HIF-1α nuclear accumulation. Restoration of miR-29b level by miR-29b-3p mimic transfection abolished Nano-Ni-induced MMP-2 and MMP-9 activation and down-regulation of tight junction-associated proteins. In summary, our results demonstrated that Nano-Ni-induced dysregulation of tight junction-associated proteins in skin keratinocytes was via HIF-1α/miR-29b/MMPs pathway.

**Supplementary Information:**

The online version contains supplementary material available at 10.1186/s12989-021-00405-2.

## Background

The increasing use of nanotechnology in industry and the continuous emergence of nano-scale metal materials have brought issues related to toxicology and occupational and environmental health to the forefront. Nanoparticle exposure has been associated with a variety of human diseases, including autoimmune diseases, lung inflammation, cardiopulmonary system damage, skin damage, and neurodegenerative changes [1–4]. Compared with larger-sized particles of the native material, nanoparticles have distinct physical properties, such as high surface energy, high surface area, low melting point, low burning point, etc., that alter their toxicity profile and the approach to risk assessment and exposure control.

Nickel is widely used in industry and is the main sensitizer of occupational contact dermatitis in most industrialized countries [5]. Allergic contact dermatitis (ACD) is a common skin condition caused by a type IV delayed-type hypersensitivity response to antigens that encounter the skin. Contact dermatitis accounts for 70–90% of occupational skin disease cases [6]. Although the EU (European Union) Nickel Directive was implemented in the 1990s, nickel contact allergy still affects 8–18% of the population [7]. Recently, a retrospective cross-sectional analysis enrolling over 40,000 patients in North America who underwent patch testing showed that 17.2% of the general population had nickel sensitivity, 18.2% had an occupation-related nickel allergy, and 23.7% of the pediatric population had nickel sensitivity [8–10]. Individuals are at risk for multiple routes of occupational or non-occupational exposure, including dermal, oral, or transnasal. Our previous studies showed that exposure to nickel nanoparticles (Nano-Ni) caused severe and persistent lung inflammation and fibrosis, which were strongly linked to pulmonary toxicity [11–14]. Case reports of occupational Nano-Ni exposure illustrate potential health risks. Accidental exposure to a high concentration of Nano-Ni by spraying Nano-Ni onto bushes for turbine bearings using a metal arc process resulted in the worker dying of adult respiratory distress syndrome (ARDS) at day 13 post-exposure [15]. A chemist developed new skin reactions to earrings and belt buckles after she began working with nickel nanoparticle powder without protective measures [16]. The potential for Nano-Ni to induce morbidity and mortality makes understanding the underlying mechanisms a priority to safeguard the public and to develop countermeasures.

The skin is the first barrier against pathogens and allergens. Destruction of the epidermal barrier sensitizes the local skin to allergens and accelerates the penetration of haptens. The stratum corneum and tight junctions are two important structures that contribute to this barrier. Tight junctions in the stratum granulosum seal the layer from the external environment. Tight junctions are composed of transmembrane proteins, i.e., claudins, occludin, tricellulin, and junctional adhesion molecules as well as tight junction plaque proteins [17]. Accumulating evidence indicates that tight junctions play important and diverse roles in the progression of allergic dermatitis [18]. Previous studies indicated that claudin 1 - deficient mice would die within 24 h of birth from severe dehydration, which suggested the indispensable role of tight junctions in skin homeostasis [19]. In atopic dermatitis subjects, researchers observed barrier dysfunction and immune dysregulation caused by the impairment of tight junctions [20]. Previous studies also showed that exposure to *p*-phenylenediamine led to the downregulation of tight junction and stratum corneum proteins in both asymptomatic patients and patients with mild acute contact dermatitis [21]. Certain metal particles, such as nickel, cobalt, chromium, and zinc, which are ubiquitous in our environment, may result in allergic contact dermatitis and systemic contact dermatitis. Systemic reactions, such as hand dermatitis or generalized eczematous reactions, can occur due to dietary nickel or cobalt ingestion. Furthermore, the unique properties of nanoparticles made from the bulk material may result in additional health effects. Skin exposure to nanoparticles raises concerns over their direct dermatologic toxicities and their transdermal toxicities, but current understanding of their toxicities and immune responses remains insufficient. Exposure to silver and silica nanoparticles has been shown to dysregulate tight junction-associated proteins in the brain endothelial cells or lung epithelial cells [22, 23]. However, few studies focused on the effects of Nano-Ni on tight junction-associated proteins in epidermal keratinocytes.

Matrix metallopeptidases (MMPs) are a family of structurally and functionally related zinc endopeptidases, which are capable of degrading the components of the extracellular matrix and basement membrane, including collagens, proteoglycans, fibronectin, laminin, et al. [24, 25]. Over the past decades, MMP research has gained considerable attention in various pathologies, such as inflammation [26], auto-immune diseases [27], angiogenesis [28], neuropathological disorders [29], tumor progression [30], and so on. Among the MMPs, the 72 kDa gelatinase A (or MMP-2) and the 92 kDa gelatinase B (or MMP-9) are widely expressed in the body by different cell types [31], and both gelatinases are involved in the alteration of the epidermal architecture of the lesions. In allergic contact dermatitis patients, MMP-2 and MMP-9 expression was significantly increased in involved skin compared to uninvolved skin [32]. The expression of these gelatinases also tended to increase temporarily in the acute phase of chronic contact dermatitis in mouse skin [33]. Previous studies have demonstrated that matrix metalloproteinases are involved in the disruption of tight junction proteins [34–36], which may affect the epidermal barrier. Our previous studies also showed that exposure to cobalt or nickel nanoparticles caused increased expression and activity of MMP-2 and MMP-9. This raised the intriguing possibility that Nano-Ni-induced MMPs production may be involved in Nano-Ni-induced disruption of tight junction-associated proteins.

MicroRNAs (miRNAs) are a class of non-coding single-stranded RNA molecules of ~ 22 nucleotides in length. miRNA mainly binds to the complementary seed sequence in the untranslated region (3′-UTR) of the target mRNA under the guidance of the RNA-induced silencing complex, thereby promoting mRNA degradation or inhibiting protein translation. Since the discovery of the first miRNA in *Caenorhabditis elegans*, the field of miRNA biology has greatly expanded [37]. Accumulating evidence indicates that miRNAs are involved in various skin disorders, including allergic drug eruptions [38, 39], atopic dermatitis [40, 41], and delayed-type hypersensitivity reaction [42, 43]. miR-29b is a member of the miR-29 family and was previously reported as a tumor suppressor by regulating the tumor microenvironment [44, 45]. It was reported that increased miR-29b expression inhibited the proliferation and migration of human skin fibroblasts after thermal injury by targeting collagen 1 [46]. miR-29b also participates in diabetic would healing by regulating the biological function of endothelial cells [47]. Moreover, Remlarsen, a miR-29b mimic, was effective in preventing the formation of a fibrotic scar [48]. Previous studies also showed that miR-29 family members may be involved in Nrf2-induced structural alteration of epidermal desmosomes [49]. This collection of results suggested a potential role of miR-29b in skin disorders. However, few studies investigated the role of miR-29 on the tight junction-associated proteins in keratinocytes exposed to metal nanoparticles.

In the present study, we examined whether exposure to Nano-Ni altered tight junction-associated proteins in human skin keratinocytes and the potential mechanisms underlying these effects. We hypothesized that exposure of HaCaT cells to Nano-Ni would cause dysregulation of tight junction-associated proteins via the HIF-1α/miR-29b/MMPs axis. We first investigated whether exposure to Nano-Ni induced alteration of the transcription and activity of MMP-2 and MMP-9, and the expression of tight junction-associated proteins (claudin-1, occludin, and ZO-1). We then evaluated HIF-1α nuclear accumulation and miR-29b expression in HaCaT cells after exposure to Nano-Ni. The role of HIF-1α in Nano-Ni-induced dysregulation of miR-29b and MMPs was explored by using a HIF-1α inhibitor, CAY10585. Bioinformatic analysis and previous literature have shown that miR-29b-3p targets both MMP-2 and MMP-9 [45]. Thus, we further explored whether dysregulation of miR-29b was involved in Nano-Ni-induced upregulation of MMP-2 and MMP-9, which might lead to dysregulation of tight junction-associated proteins. In this study, titanium dioxide nanoparticles (Nano-TiO_2_) were used as a control since our and other previous studies have shown that exposure to Nano-TiO_2_ did not cause HIF-1α nuclear accumulation and MMP-2/9 upregulation in human monocytes [50, 51], and topical exposure to Nano-TiO_2_ did not induce acute cutaneous irritation or skin sensitization [52, 53].

## Methods

### Metal nanoparticles and their characterization

Nano-Ni and Nano-TiO_2_ with a mean diameter of 20 nm and 28 nm were obtained from Inabata & Co., Ltd., Vacuum Metallurgical Co., Ltd., Japan. The characterization of these nanoparticles was described in our previous studies [11–14, 50]. Briefly, the specific surface area is 43.8 m^2^/g for Nano-Ni and 45.0 m^2^/g for Nano-TiO_2_. Nano-Ni is composed of 85–90% of metal nickel and 10–15% of NiO; Nano-TiO_2_ is composed of 90% of anatase and 10% of rutile. The size of particles and agglomerates in the cell culture medium is 250 nm for Nano-Ni and 280 nm for Nano-TiO_2_ [11, 50]. The Zeta potential is 2.0 ± 1.4 mV for Nano-Ni [11]. The solubility of Nano-Ni is 16.48 ± 0.58 (ppm) in 1x PBS and 81.23 ± 3.76 (ppm) in the cell culture medium, and Nano-TiO_2_ is less than 1 ppm both in 1x PBS and cell culture medium [50]. Nano-Ni and Nano-TiO_2_ were dispersed in 1x PBS, ultrasonicated for 10 min, and vibrated thoroughly prior to each experiment.

### Chemicals and reagents

Anti-HIF-1α antibody (cat. no. 610959) was obtained from BD (San Jose, CA, USA); anti-ZO-1 antibody (cat. no. bs-1329R) from Bioss Antibodies (Woburn, MA, USA); anti-occludin antibody (cat. no. NBP1–77037) from Novus Biologicals (Littleton, CO, USA); anti-claudin-1 antibody (cat. no. 13255) and anti-β-Actin antibody (cat. no. 58169) from Cell Signaling Technology (Beverly, MA, USA). Gelatin was purchased from Acros Organics (Morris Plains, NY, USA). CAY10585 was obtained from BioVision (Milpitas, CA, USA). The *mir*Vana® miRNA mimic (has-miR-29b-3p, cat. no. MC10103), Negative Control #1, and Lipofectamine™ RNAiMAX Transfection Reagent were purchased from Thermo Fisher Scientific (NY, USA). All other chemicals were purchased from Fisher (Pittsburgh, PA, USA) unless otherwise indicated. All chemicals used were of analytic grade.

### Cell culture and treatment

Immortalized adult human skin keratinocytes HaCaT were originally established at the German Cancer Research Center (DKFZ) and obtained from CLS Cell Lines Service GmbH (Eppelheim, German). HaCaT cells were routinely cultured in Dulbecco’s modified Eagle’s medium (DMEM, Mediatech, Inc., Manassas, VA, USA) supplemented with 10% fetal bovine serum (Mediatech), 100 U/mL penicillin, and 100 μg/mL streptomycin (Mediatech) at 37 °C in a humidified atmosphere with 5% CO_2_. HaCaT cells were treated with different doses of Nano-Ni or Nano-TiO_2_ for different times. For MMP-2 and MMP-9 activity analysis, cells were cultured in serum-free DMEM for 24 h to minimize the selective activation of MMPs. To explore the effects of HIF-1α on MMP-2 and MMP-9, cells were pre-treated with CAY10585 (30 μM) for 2 h prior to exposure to Nano-Ni. For transfection, 1 × 10^5^ cells were seeded in each well of 6-well plates (Corning Inc., Corning, NY, USA) and maintained overnight. The specific hsa-mir-29b-3p mimic or negative control were then transfected into cells by using Lipofectamine™ RNAiMAX Transfection Reagent according to the manufacturer’s protocol. Then the cells were submitted to Nano-Ni or Nano-TiO_2_ exposure and for further functional analysis. Unexposed HaCaT cells were used as negative controls.

### Cytotoxicity assays

The cytotoxicity of Nano-Ni or Nano-TiO_2_ on HaCaT cells was determined by CellTiter 96® AQ_ueous_ Non-Radioactive Cell Proliferation Assay (MTS assay; Promega, WI, USA) and In Vitro Toxicity Assay Kit, Sulforhodamine B based (SRB assay; Sigma, MO, USA) according to the manufacturer’s instructions. For the MTS assay, 1 × 10^4^ HaCaT cells in 100 μL medium were seeded into each well of a 96-well plate, and allowed to attach to the growth surface by culturing overnight. The cells were then treated with different doses of Nano-Ni or Nano-TiO_2_ for 24 h. Afterward, 20 μL of MTS/PMS mixture (20:1) was directly added to each well of the plate and cells were incubated in the incubator for 3 h. The absorbance was measured using the multidetection microplate reader (Synergy HT, BioTek, Vermont, USA) at a wavelength of 490 nm. The cell viability of the experimental cultures was calculated as a percentage of cell viability in control cultures. For SRB assay, HaCaT cells were plated into 96-well plates at a density of 1 × 10^4^ cells/100 μL and treated with Nano-Ni or Nano-TiO_2_ for 24 h as mentioned above. A total of 50 μL cold 50% TCA (trichloroacetic acid) was added to each well to fix the cells for 1 h. After the cells were rinsed with H_2_O for 4 ~ 5 times and dried at room temperature (RT), 50 μL 0.4% SRB (Sulforhodamine B Solution) was added to cover the culture surface area and set at RT for 30 min. The cells were then washed with 1% acetic acid 5 times, dried at RT, added with 100 μL 10 mM Tris, and shook for 5 min. The background absorbance was measured at a wavelength of 690 nm and subtracted from the measurement at 565 nm.

### RNA extraction and quantitative real-time PCR

Total RNA was extracted using TRIzol reagent (Sigma-Aldrich, St Louis, MO, USA) according to the manufacturer’s instructions. The concentration and quality of the RNA were determined using a spectrophotometer (Beckman Coulter, Fullerton, CA, USA). Complementary DNA (cDNA) of genes were reverse-transcribed from total RNA using M-MLV reverse transcriptase (Promega, Madison, WI, USA) and oligo (dT)_18_ (Sigma), and the iTaq™ Universal SYBR® Green Supermix (Bio-Rad, Hercules, CA, USA) was used for amplification. The cDNA of miR-29b-3p was synthesized via the TaqMan® MicroRNA Reverse Transcription Kit (Thermo Fisher Scientific), and the TaqMan® Universal Master Mix II was applied for amplification. PCR analysis was performed by the BioRad iQ5 Multicolor Real-Time PCR Detection System (Bio-Rad). Data were quantified by the 2^−ΔΔCt^ (Livak) method [54]. The PCR reaction was performed as follows: 35 cycles at 94 °C for 45 s, at 62 °C for 45 s, and at 72 °C for 45 s for MMP-2 and MMP-9; 35 cycles at 94 °C for 45 s, at 58.5 °C for 45 s, and at 72 °C for 45 s for TIMP-1, TIMP-2, and β-actin. The primers for human MMP-9 were: forward 5′-CGG TGA TTG ACG ACG CCT TTG C-3′ and reverse 5′-CGC TGT CAA AGT TCG AGG TGG TA-3′; for human MMP-2 were: forward 5′-ATT TGG CGG ACT GTG ACG-3′ and reverse 5′-GCT TCA GGT AAT AGG CAC-3′; for human TIMP-1 were: forward 5′-AAT TCC GAC CTC GTC ATC AG-3′ and reverse 5′-GTT TGC AGG GGA TGG ATA AA-3′; for human TIMP-2 were: forward 5′-CTG GAC GTT GGA GGA AAG AA-3′ and reverse 5′-GTC GAG AAA CTC CTG CTT GG-3′; and for human β-actin were: forward 5′-CAT CGA GCA CGG CAT CGT CA-3′ and reverse 5′-TAG CAC AGC CTG GAT AGC AAC-3′.

### Gelatin zymography assay

MMP-2 and MMP-9 activities were measured by gelatin zymography assay as previously described [11, 12, 50, 51, 55, 56]. Briefly, HaCaT cells were cultured in 6-well plates in DMEM without FBS for 24 h before exposure to Nano-Ni or Nano-TiO_2_. The conditioned media were collected and subjected to gelatin zymography under non-reducing conditions. Supernatant from cultured HT1080 cells served as a positive control, which has been described elsewhere [50, 51, 55, 56]. After electrophoresis on 10% SDS-polyacrylamide gel including 0.5 mg/mL gelatin, the gels were washed twice (30 min each) in 50 mM Tris-HCl (pH 7.5) containing 2.5% Triton X-100 (Fisher, Fair Lawn, NJ), followed by incubating in development buffer (pH 7.5) consisting of 0.2 M NaCl, 7.55 mM CaCl_2_, 1 μM ZnCl_2_, and 1% Triton X-100 (Fisher) at 37 °C overnight. After staining with 0.1% Coomassie Brilliant Blue R-250 (Bio-Rad, Hercules, CA) in 10% acetic acid and 50% methanol for 1 h with gentle agitation, gels were destained in 10% acetic acid until the clear bands were observed against the background of Coomassie Brilliant Blue-stained gel. The signal intensity of the band was quantified by ImageJ software (http://imagej.nih.gov/ij/).

### Protein extraction and Western blot

Total protein was extracted via RIPA buffer (Santa Cruz, CA) for the measurement of tight junction-associated proteins, and nuclear protein was extracted using NE-PER® Nuclear and Cytoplasmic Extraction Reagent (Thermo Fisher Scientific, Rockford, IL) to detect the alteration of HIF-1α expression after HaCaT cells were exposed to Nano-Ni or Nano-TiO_2_. The protein concentration was determined by the Bradford method using a spectrophotometer (Beckman Coulter, Brea, CA). 30 μg total protein or 20 μg nuclear protein of each sample was separated on 10% or 7.5% SDS-PAGE and transferred to polyvinylidene difluoride (PVDF) membranes (Bio-Rad). After blocking with 5% skim milk/TBST (50 mM Tris-HCl, 140 mM NaCl, 0.05% Tween-20, pH 7.5) for 2 h at room temperature, the membranes were then incubated with primary antibodies at 4 °C overnight. After washing, the membranes were incubated with horse anti-mouse or goat anti-rabbit secondary antibody conjugated with horseradish peroxidase (HRP) at room temperature for 2 h. Immunoreactive bands were detected using SuperSignal™ West Pico PLUS Chemiluminescent Substrate (Thermo Scientific, Rockford, IL) followed by exposure to CL-XPosure™ film (Thermo Scientific). Equal protein loading was verified by Coomassie Brilliant Blue staining. The expression of β-actin was used as an internal reference. Immunoreactive bands were quantified by NIH ImageJ software (http://imagej.nih.gov/ij/).

### Immunofluorescent staining

Immunofluorescent staining was performed based on the methods described previously [57–60]. Briefly, HaCaT cells were seeded into each chamber of 4-well LAB-TEK® II chamber slides (Nalge Nunc International, IL, USA) and incubated overnight to allow the cells to attach to the slide surface. After exposure to 20 μg/mL of Nano-Ni or Nano-TiO_2_ for 24 h, the cells were fixed in 10% neutral buffered formalin for 20 min and rinsed with 1x PBS three times for 5 min each. The cells were then incubated with a blocking solution (3.26% of BSA, 5% of normal goat serum, and 0.3% Triton X-100) for 60 min at room temperature for cell permeabilization and blocking of nonspecific protein binding. After incubation with anti-ZO-1 (1: 500) overnight at 4 °C and washed with 1x PBS for three times, the cells were incubated with Alexa Fluor® 488-conjugated goat anti-rabbit IgG (Invitrogen, Carlsbad, CA, USA) for 60 min at room temperature. After washing, the slides were mounted with Prolong Gold Antifade Reagent with DAPI (Invitrogen) and examined under fluorescence microscopy (Nikon, Japan). At cell-to-cell contacts, the ZO-1 protein is located on the cytoplasmic membrane surface of intercellular tight junctions. This appears as a linear or gasket-like pattern after immunofluorescent staining. To ensure the comparability of ZO-1 protein fluorescence after multiple treatments, the exposure time of ZO-1 fluorescence (green) was set to 100 ms, and the DAPI fluorescence (blue) was set to 5 ms for all groups. ZO-1 immunofluorescence intensity was quantified by NIH ImageJ software.

### Statistical analysis

SigmaPlot 13.0 software (Systat Software, San Jose, CA, USA) was used for data management and statistical analyses. The data were presented as mean ± SE. Each experiment was performed at least three times. A comparison of two groups was carried out by the *t*-test (for example, comparison of the Nano-Ni and Nano-TiO_2_ effects at the same dose). If there were more than two groups (for example, dose- or time-dependent study), differences among groups were evaluated by one-way analysis of variance (ANOVA). When ANOVA showed an overall difference, Dunnett’s *t*-test was performed. If there were two independent variables on a dependent variable (for example, with/without Nano-Ni and HIF-1α inhibitor CAY10585 treatment), two-way ANOVA was employed, with the Nano-Ni group being the first factor and others, such as the HIF-1α inhibitor CAY10585, as the second factor. Should the two-way ANOVA demonstrate significant differences, a Holm-Sidak test was used for multiple comparisons. If necessary, transformation of the data was used to achieve normally distributed data before ANOVA analysis. *P* < 0.05 was considered statistically significant.

## Results

### Cytotoxic effects of Nano-Ni and Nano-TiO_2_ on HaCaT cells

**Fig. 1:**
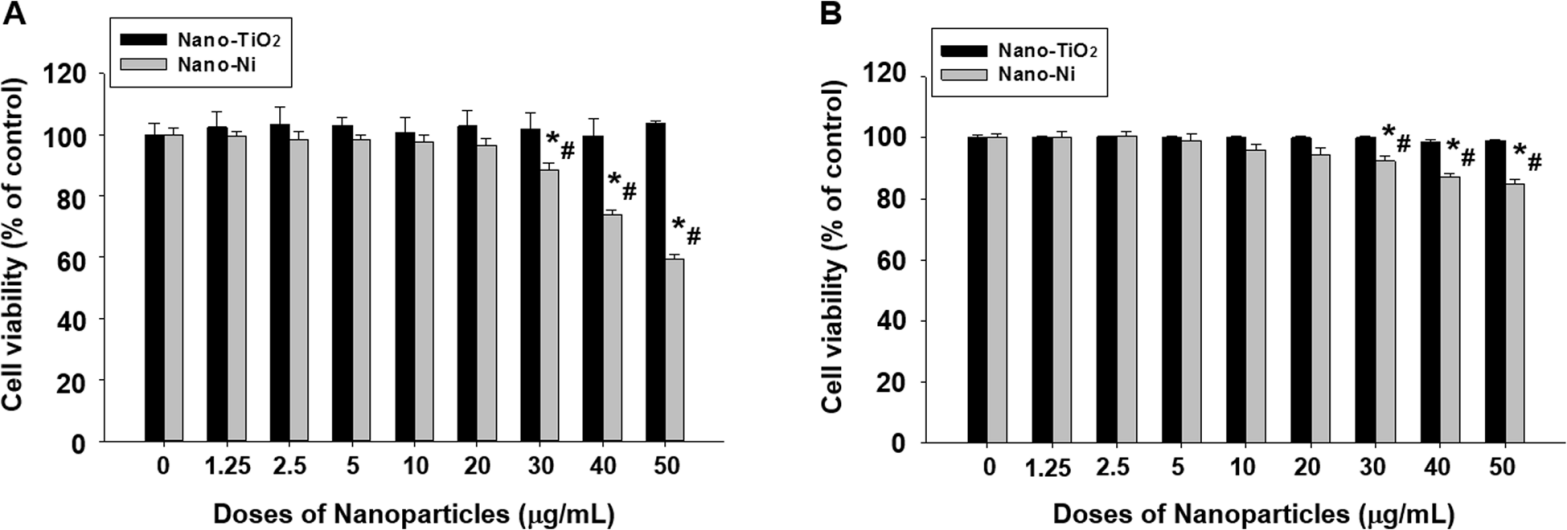
Cytotoxicity of Nano-Ni and Nano-TiO_2_ in HaCaT cells. HaCaT cells were seeded into 96-well plates and treated with different concentrations of Nano-Ni or Nano-TiO_2_ for 24 h. Cells without treatment were used as control. The cytotoxicity was determined by MTS assay (**a**) and SRB assay (**b**). Data represent mean ± SE (*n* = 6). * Significant difference as compared with the control group, *p* < 0.05; ^#^ Significant difference as compared with the same dose of Nano-TiO_2_-treated group, *p* < 0.05

HaCaT cells were exposed to various concentrations of Nano-Ni or Nano-TiO_2_ for 24 h, and cell viability was measured by both CellTiter 96® AQ_ueous_ Non-Radioactive Cell Proliferation Assay (MTS assay) and In Vitro Toxicity Assay Kit, Sulforhodamine B based (SRB assay) as described above. MTS assay is a colorimetric method for determining the number of metabolically active cells in which the dehydrogenase enzymes can convert a tetrazolium compound (MTS) into an aqueous, soluble, and colored formazan. SRB assay is a means of measuring total biomass by staining cellular proteins with the Sulforhodamine B. Exposure of HaCaT cells to Nano-Ni at 30 μg/mL and beyond caused significant cell death by MTS assay, while exposure to 20 μg/mL or less of Nano-Ni did not cause significant cell death (Fig. 1a). However, exposure of HaCaT cells to any doses from 0 to 50 μg/mL of Nano-TiO_2_ did not cause any cytotoxic effects (Fig. 1a). The results were further confirmed by SRB assay (Fig. 1b). Therefore, the non-toxic doses (≤ 20 μg/mL) were selected to explore the effects of Nano-Ni on HaCaT cells in the following experiments.

### Exposure of HaCaT cells to Nano-Ni caused increased mRNA expression of MMP-2 and MMP-9 and increased activity of MMP-2 and MMP-9

**Fig. 2:**
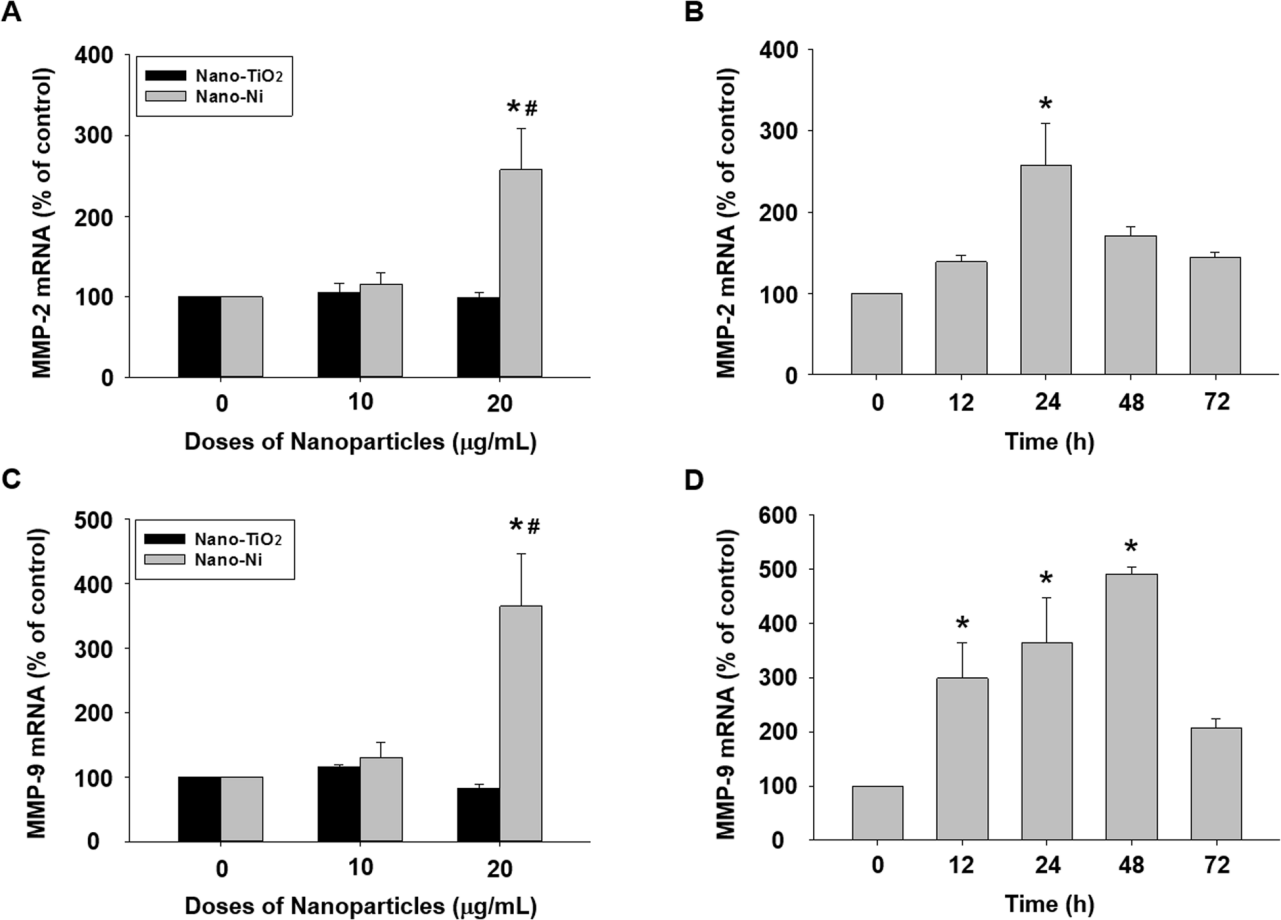
Dose- and time-dependent induction of MMP-2 and MMP-9 mRNAs in HaCaT cells exposed to Nano-Ni. Expression of MMP-2 and MMP-9 mRNA in HaCaT cells were determined by real-time PCR after cells were exposed to 10 or 20 μg/mL of Nano-Ni or Nano-TiO_2_ for 24 h (**a** & **c**), or to 20 μg/mL of Nano-Ni for 12, 24, 48, or 72 h (**b** & **d**). Cells without treatment were used as control. Data represent mean ± SE (*n* = 3). * Significant difference as compared with the control group, *p* < 0.05; ^#^ Significant difference as compared with the same dose of Nano-TiO_2_-treated group, *p* < 0.05

The effects of Nano-Ni or Nano-TiO_2_ on MMP-2 and MMP-9 mRNA expression were investigated by real-time PCR. The results showed that a remarkable increase in MMP-2 and MMP-9 mRNA levels after cells were exposed to 20 μg/mL of Nano-Ni for 24 h was observed, whereas Nano-TiO_2_ did not cause any significant change (Fig. 2a & c). In the time-response study, MMP-2 mRNA level increased significantly after 24 h of 20 μg/mL of Nano-Ni exposure, and MMP-9 mRNA level increased dramatically after 12, 24, and 48 h of exposure (Fig. 2b & d). Again, Nano-TiO_2_ did not cause any alteration of MMP-2 and MMP-9 mRNA levels (data not shown).

**Fig. 3:**
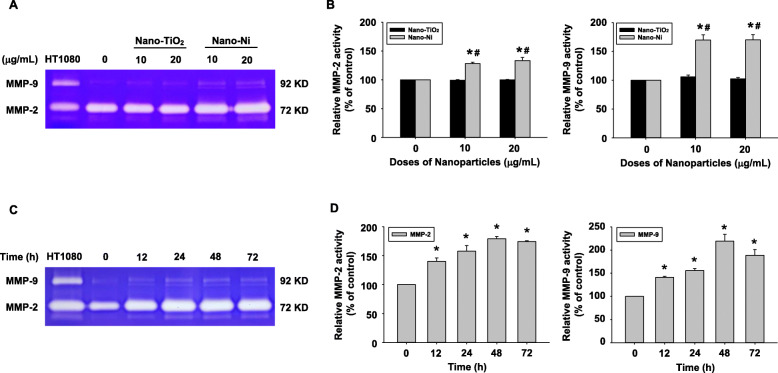
Dose- and time-dependent induction of MMP-2 and MMP-9 activities in HaCaT cells exposed to Nano-Ni. HaCaT cells were exposed to 10 or 20 μg/mL of Nano-Ni or Nano-TiO_2_ for 24 h (**a** & **b**), or to 20 μg/mL of Nano-Ni for 12, 24, 48, or 72 h (**c** & **d**). Cells without treatment were used as control. MMP-2 and MMP-9 activities were determined in the conditioned media by gelatin zymography assay. A and C are the results of a single experiment, while B and D are normalized band densitometry readings averaged from three independent experiments ± SE. Supernatants collected from serum-free cultured HT1080 cells were used as a molecular weight marker for MMP-2 and MMP-9. * Significant difference as compared with the control group, *p* < 0.05; ^#^ Significant difference as compared with the same dose of Nano-TiO_2_-treated group, *p* < 0.05

The activities of MMP-2 and MMP-9 in HaCaT cells after Nano-Ni or Nano-TiO_2_ exposure were determined by gelatin zymography assay. A dose-response increase in MMP-2 and MMP-9 activity was observed in the conditioned media from HaCaT cells exposed to 10 and 20 μg/mL of Nano-Ni for 24 h (Fig. 3a & b). In the time-response study, the results showed that exposure of HaCaT cells to 20 μg/mL of Nano-Ni for 12, 24, and 48 h caused a significant increase in MMP-2 and MMP-9 activities as compared with those in the control, and their activities peaked at 24 h after Nano-Ni exposure (Fig. 3c & d). Consistent with the qRT-PCR results, exposure of HaCaT cells to Nano-TiO_2_ did not cause any dose-response (Fig. 3a & b) or time-response (data not shown) changes in the MMP-2 and MMP-9 activities.

**Fig. 4:**
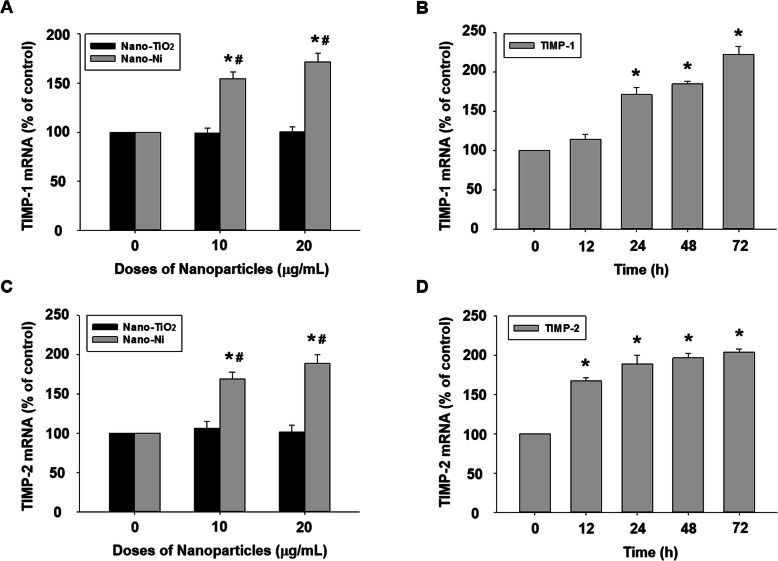
Dose- and time-dependent induction of TIMP-1 and TIMP-2 mRNAs in HaCaT cells exposed to Nano-Ni. Expression of TIMP-1 and TIMP-2 mRNAs in HaCaT cells were determined by real-time PCR after cells were exposed to 10 or 20 μg/mL of Nano-Ni or Nano-TiO_2_ for 24 h (**a** & **c**), or 20 μg/mL of Nano-Ni for 12, 24, 48, or 72 h (**b** & **d**). Cells without treatment were used as control. Data represent mean ± SE (*n* = 3). * Significant difference as compared with the control group, *p* < 0.05; ^#^ Significant difference as compared with the same dose of Nano-TiO_2_-treated group, *p* < 0.05

TIMP-1 and TIMP-2 are endogenous tissue inhibitors of metalloproteinases [61]. The mRNA expression of TIMP-1 and TIMP-2 was also measured in HaCaT cells treated with Nano-Ni. The real-time PCR results indicated that exposure of HaCaT cells to Nano-Ni (10 and 20 μg/mL) for 24 h significantly enhanced mRNA levels of TIMP-1 and TIMP-2, which was in a dose-dependent manner (Fig. 4a & c). However, Nano-TiO_2_ exposure had no effects on TIMP-1 and TIMP-2 mRNA expression (Fig. 4a & c). TIMP-1 and TIMP-2 mRNA expression levels remarkably increased in HaCaT cells exposed to 20 μg/mL of Nano-Ni for 12, 24, 48, or 72 h (Fig. 4b & d).

### Nano-Ni exposure caused down-regulation of tight junction-associated proteins

**Fig. 5:**
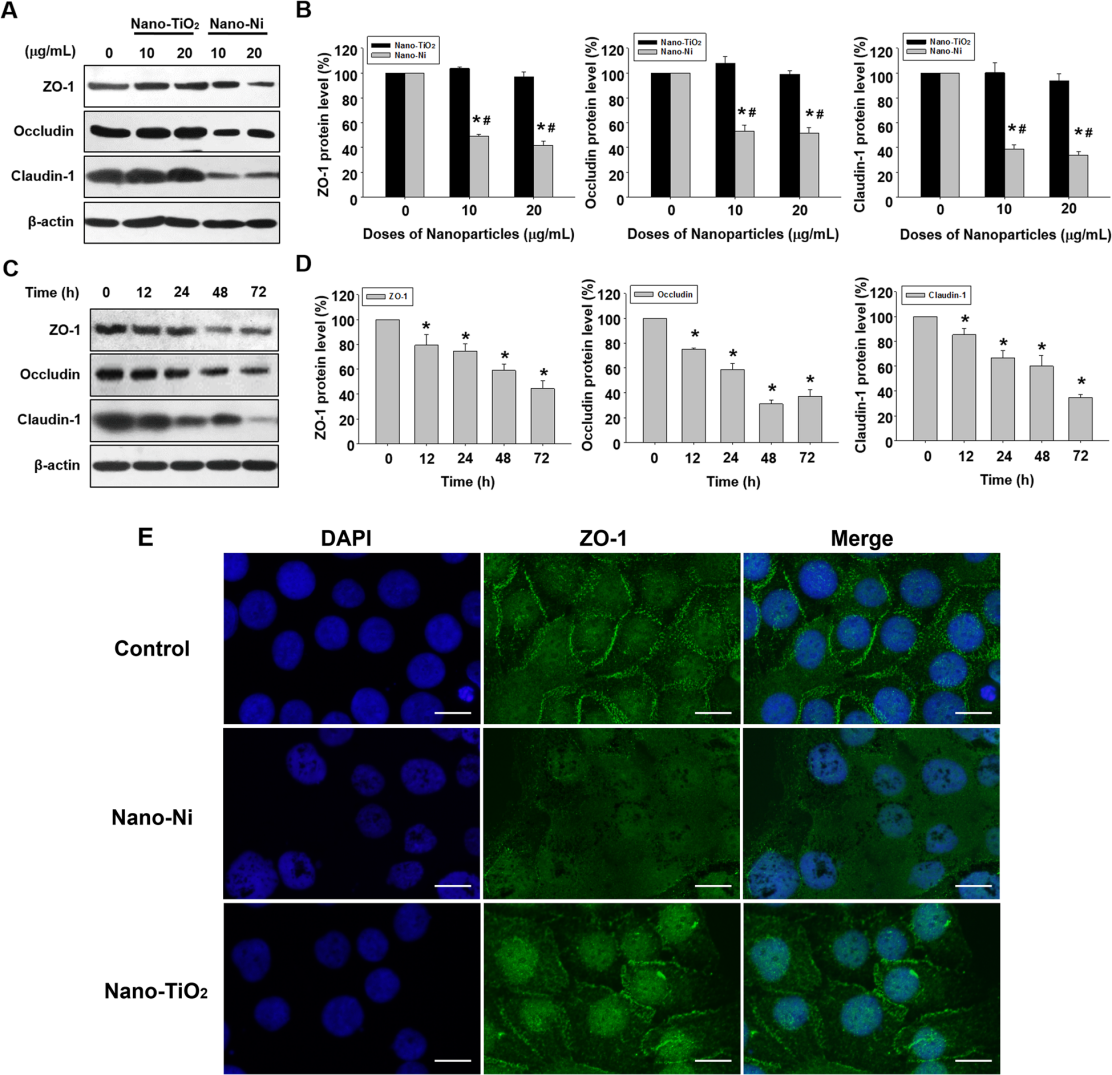
Dose- and time-dependent reduction of tight junction-associated proteins in HaCaT cells exposed to Nano-Ni. **a**-**d** Expression of tight junction-associated proteins in HaCaT cells were determined by Western blot after cells were exposed to 10 or 20 μg/mL of Nano-Ni or Nano-TiO_2_ for 24 h (**a** & **b**), or to 20 μg/mL of Nano-Ni for 12, 24, 48, or 72 h (**c** & **d**). Cells without treatment were used as control. **a** and **c** are the results of a single experiment, while **b** and **d** are normalized band densitometry readings averaged from three independent experiments ± SE. * Significant difference as compared with the control group, *p* < 0.05; ^#^ Significant difference as compared with the same dose of Nano-TiO_2_-treated group, *p* < 0.05. **e** Downregulation of ZO-1 (green staining) in HaCaT cells exposed to 20 μg/mL of Nano-Ni for 24 h by immunofluorescent staining. DAPI (blue) stains the whole nucleus of a cell. Scale bar represents 20 μm for all panels

To determine whether exposure to metal nanoparticles would cause expression alteration of tight junction-associated proteins (ZO-1, occludin, and claudin-1), HaCaT cells were treated with 0, 10, and 20 μg/mL of Nano-Ni or Nano-TiO_2_ for 24 h and the expression of tight junction-associated proteins were measured by Western blot. A significant reduction in ZO-1, occludin, and claudin-1 expression was observed in HaCaT cells exposed to 10 and 20 μg/mL of Nano-Ni, but not Nano-TiO_2_ (Fig. 5a & b). The time-response study showed that the expression levels of these tight junction-associated proteins significantly decreased in cells treated with 20 μg/mL of Nano-Ni for 12, 24, 48, or 72 h (Fig. 5c & d). The ZO-1 expression in HaCaT cells was also examined by immunofluorescent staining. The results showed that exposure of cells to 20 μg/mL of Nano-Ni for 24 h caused decreased expression of ZO-1, while exposure to Nano-TiO_2_ did not (Fig. 5e & Additional file 1).

### Nano-Ni exposure caused HIF-1α nuclear accumulation in HaCaT cells

**Fig. 6:**
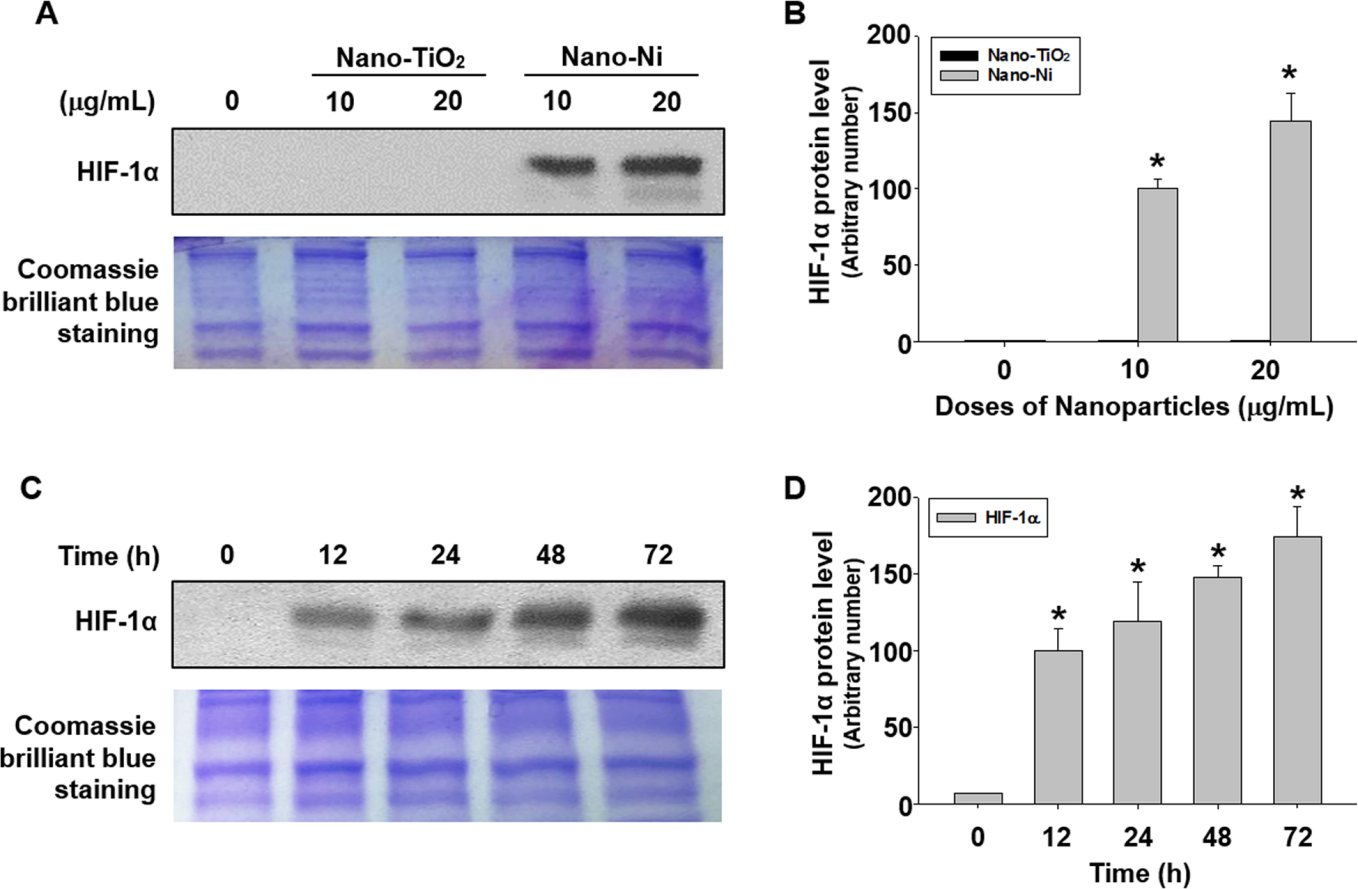
HIF-1α nuclear accumulation in HaCaT cells exposed to Nano-Ni. Expression of HIF-1α in HaCaT cells was determined by Western blot after cells were exposed to 10 or 20 μg/mL of Nano-Ni or Nano-TiO_2_ for 24 h (**a**), or to 20 μg/mL of Nano-Ni for 12, 24, 48, or 72 h (**c**). The representative results of three Western blot experiments are shown. Cells without treatment were used as control. Equal protein loading was verified by Coomassie Brilliant Blue staining. **b** & **d** are normalized band densitometry readings averaged from three independent experiments ± SE. * Significant difference as compared with the control group, *p* < 0.05

HIF-1α nuclear accumulation after Nano-Ni or Nano-TiO_2_ exposure was determined by Western blot. The results showed that exposure of cells to 10 and 20 μg/mL of Nano-Ni for 24 h caused a marked enhancement of HIF-1α nuclear accumulation (Fig. 6a & b). However, HIF-1α expression was not significantly different from the background in the control and Nano-TiO_2_-treated groups. A significant nuclear accumulation of HIF-1α in HaCaT cells exposed to 20 μg/mL of Nano-Ni for 12, 24, 48, and 72 h was also observed (Fig. 6c & d).

### Nano-Ni exposure caused down-regulation of miR-29b in HaCaT cells

**Fig. 7:**
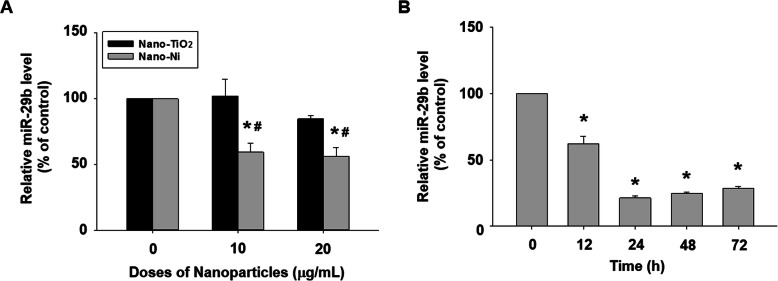
Downregulation of miR-29b in HaCaT cells exposed to Nano-Ni. The level of miR-29b in HaCaT cells was determined by real-time PCR after cells were exposed to 10 or 20 μg/mL of Nano-Ni or Nano-TiO_2_ for 24 h (**a**), or to 20 μg/mL of Nano-Ni for 12, 24, 48, or 72 h (**b**). Cells without treatment were used as control. Data represent mean ± SE (*n* = 3). * Significant difference as compared with the control group, *p* < 0.05; ^#^ Significant difference as compared with the same dose of Nano-TiO_2_-treated group, *p* < 0.05

miR-29b is known to be downregulated under hypoxic conditions [62–64]. Since a significant nuclear accumulation of HIF-1α in HaCaT cells after Nano-Ni exposure was observed in this study, we further investigated whether Nano-Ni would cause dysregulation of miR-29b in HaCaT cells. The results of the dose-response study showed that miR-29b level dramatically decreased in HaCaT cells after exposure to 10 and 20 μg/mL of Nano-Ni for 24 h (Fig. 7a). No change of miR-29b level was observed in cells with Nano-TiO_2_ treatment (Fig. 7a). The results of the time-response study demonstrated that exposure of HaCaT cells to 20 μg/mL of Nano-Ni for 12, 24, 48, and 72 h caused a marked decrease in miR-29b expression (Fig. 7b).

### HIF-1α inhibition abolished Nano-Ni-induced dysregulation of miR-29b, MMP-2, and MMP-9

**Fig. 8:**
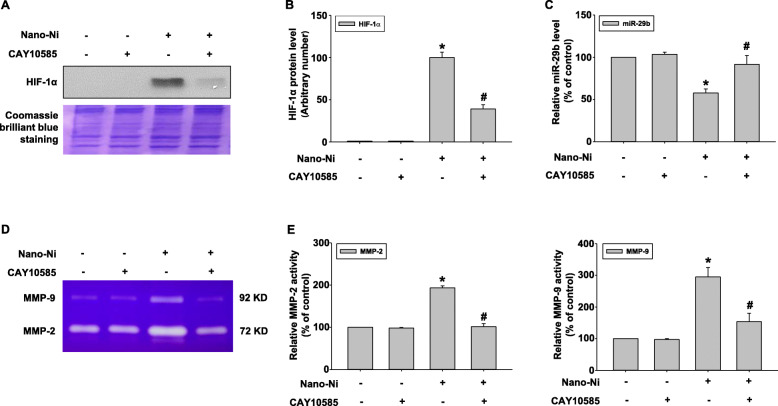
Inhibition of HIF-1α abolished Nano-Ni-induced dysregulation of miR-29b, MMP-2, and MMP-9. HaCaT cells were pretreated with 30 μM of CAY10585, a HIF-1α inhibitor, for 2 h prior to exposure to 20 μg/mL of Nano-Ni for another 24 h. Cells without treatment were used as control. HIF-1α expression was determined by Western blot and a representative result of three Western blot experiments is shown (**a**). **b** is normalized band densitometry readings averaged from three independent experiments ± SE. miR-29b expression was determined by real-time PCR (**c**), while MMP-2 and MMP-9 activities by gelatin zymography assay (**d** & **e**). Data represent mean ± SE (*n* = 3). * Significant difference as compared with the control group, *p* < 0.05; ^#^ Significant difference as compared with the group with Nano-Ni treatment, but without CAY10585 treatment, *p* < 0.05

Our results demonstrated that Nano-Ni exposure caused significant nuclear accumulation of HIF-1α, decreased expression of miR-29b, and activation of MMP-2 and MMP-9. To investigate whether HIF-1α influenced the dysregulation of miR-29b, MMP-2, and MMP-9 in HaCaT cells after Nano-Ni exposure, a HIF-1α inhibitor, CAY10585, was used to inhibit HIF-1α accumulation. CAY10585 has been proved to promote proteasomal degradation of HIF-1α by upregulating von Hippel-Lindau (VHL) without affecting HIF-1β expression [65, 66]. Here, 30 μM of CAY10585 was used to pretreat HaCaT cells for 2 h before exposure to 20 μg/mL of Nano-Ni for another 24 h. Our results showed that CAY10585 remarkably inhibited Nano-Ni-induced HIF-1α nuclear accumulation (Fig. 8a & b). CAY10585 pretreatment also restored Nano-Ni-induced decreased expression of miR-29b in HaCaT cells by qRT-PCR (Fig. 8c), suggesting that Nano-Ni-induced HIF-1α nuclear accumulation was involved in Nano-Ni-induced down-regulation of miR-29b in HaCaT cells. Furthermore, a gelatin zymography assay was performed to explore whether HIF-1α also participated in Nano-Ni-induced up-regulation of MMP-2 and MMP-9. The results revealed that suppression of HIF-1α markedly abolished Nano-Ni-induced activation of MMP-2 and MMP-9 (Fig. 8d & e), demonstrating the involvement of Nano-Ni-induced HIF-1α nuclear accumulation in the increased activities of MMP-2 and MMP-9 in HaCaT cells exposed to Nano-Ni.

### The role of miR-29b in Nano-Ni-induced MMP-2 and MMP-9 upregulation

**Fig. 9:**
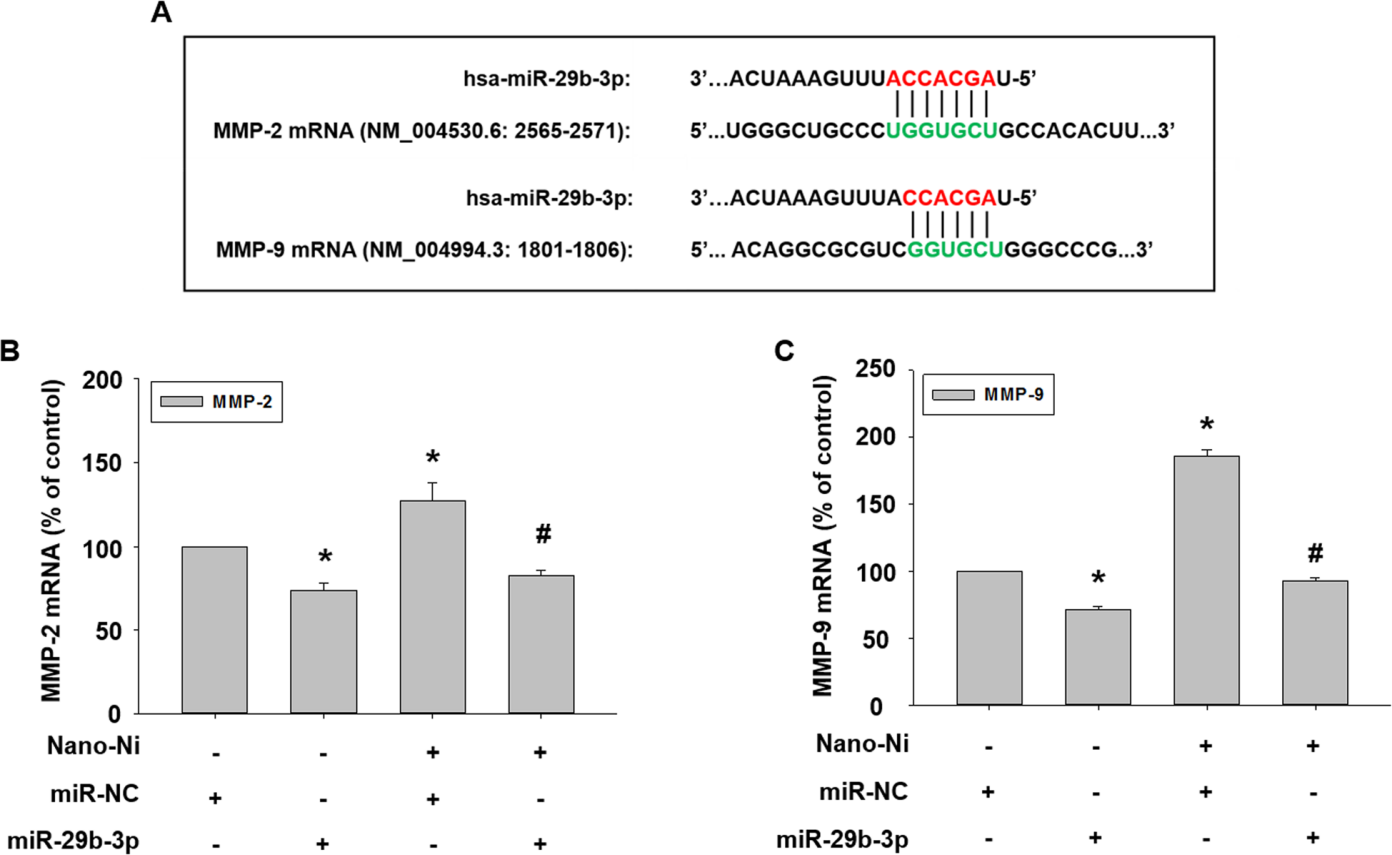
Effects of miR-29b-3p mimic on the expression of MMP-2 and MMP-9 mRNA in HaCaT cells exposed to Nano-Ni. HaCaT cells were transfected with 30 nM of miR-29b-3p mimic for 24 h, followed by 20 μg/mL of Nano-Ni treatment for another 24 h. Cells without treatment were used as control. A microRNA mimic negative control (miR-NC) was used to observe if there is any off-targeting effects of miR-29b-3p mimic. **a** shows the matching of seed sequence of miR-29b-3p with the 3′ untranslated region (3′ UTR) of both MMP-2 and MMP-9. The expression of MMP-2 and MMP-9 mRNA was determined by real time-PCR (**b** & **c**). Data represent mean ± SE (*n* = 3). * Significant difference as compared with the control group, *p* < 0.05; ^#^ Significant difference as compared with the group with Nano-Ni treatment and negative control miR-NC transfection, *p* < 0.05

To explore whether miR-29b was involved in Nano-Ni-induced upregulation of MMP-2 and MMP-9, prediction algorithms were used to screen the potential miRNAs targeting both MMP-2 and MMP-9 [45, 67, 68]. As shown in Fig. 9a, the seed sequence of miR-29b-3p was predicted to target the 3′ untranslated region (3′ UTR) of both MMP-2 and MMP-9. Thus, miR-29b-3p mimic was transfected into HaCaT cells to restore its level, which was down-regulated by Nano-Ni.

**Fig. 10:**
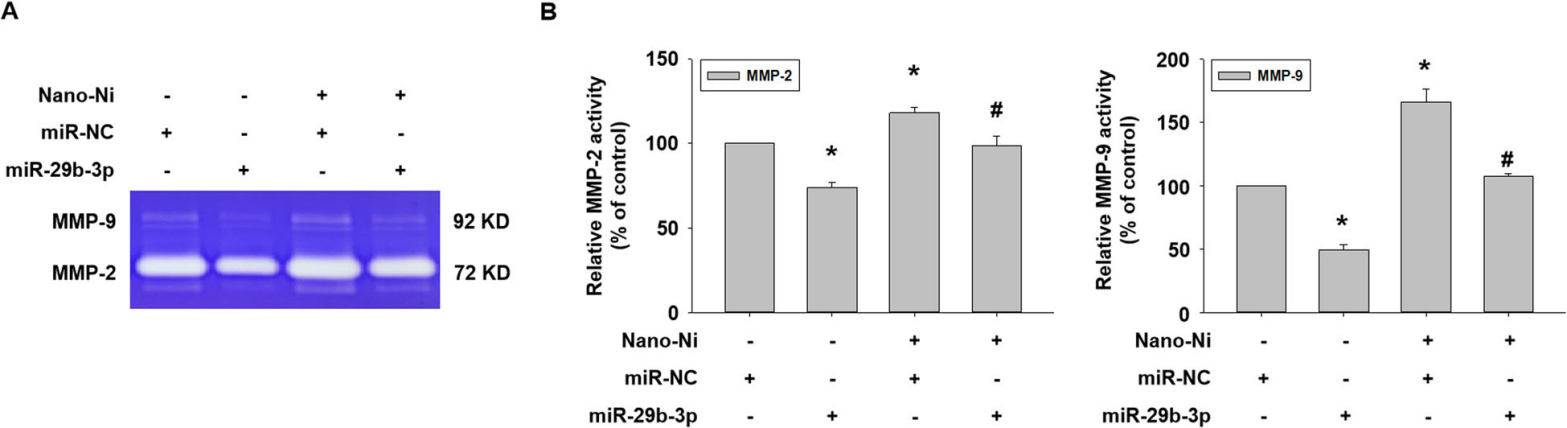
Effects of miR-29b-3p mimic on Nano-Ni-induced MMP-2 and MMP-9 activities. Conditioned medium samples were collected after HaCaT cells were transfected with miR-29b-3p mimic for 24 h prior to exposure to 20 μg/mL of Nano-Ni for another 24 h. MMP-2 and MMP-9 activities were measured by gelatin zymography assay. A microRNA mimic negative control (miR-NC) was used to see if there are any off-targeting effects of miR-29b-3p mimic. The results represent mean ± SE (*n* = 3). * Significant difference as compared with the control group, *p* < 0.05; ^#^ significant difference as compared with the group with Nano-Ni treatment and negative control miR-NC transfection, *p* < 0.05

After transfection with miR-29b-3p mimic for 24 h, cells were treated with 20 μg/mL of Nano-Ni for another 24 h. Our real-time PCR results demonstrated that the introduction of a miR-29b-3p mimic into cells significantly inhibited the endogenous MMP-2 and MMP-9 mRNA expression and abolished Nano-Ni-induced MMP-2 and MMP-9 mRNA expression (Fig. 9b & c). Activities of MMP-2 and MMP-9 in the conditioned media from cells with Nano-Ni exposure were determined by gelatin zymography assay. The results showed that transfecting a miR-29b-3p mimic into cells significantly abolished Nano-Ni-induced increased activities of MMP-2 and MMP-9 (Fig. 10a & b).

### The role of miR-29b in Nano-Ni-induced dysregulation of tight junction-associated proteins

**Fig. 11:**
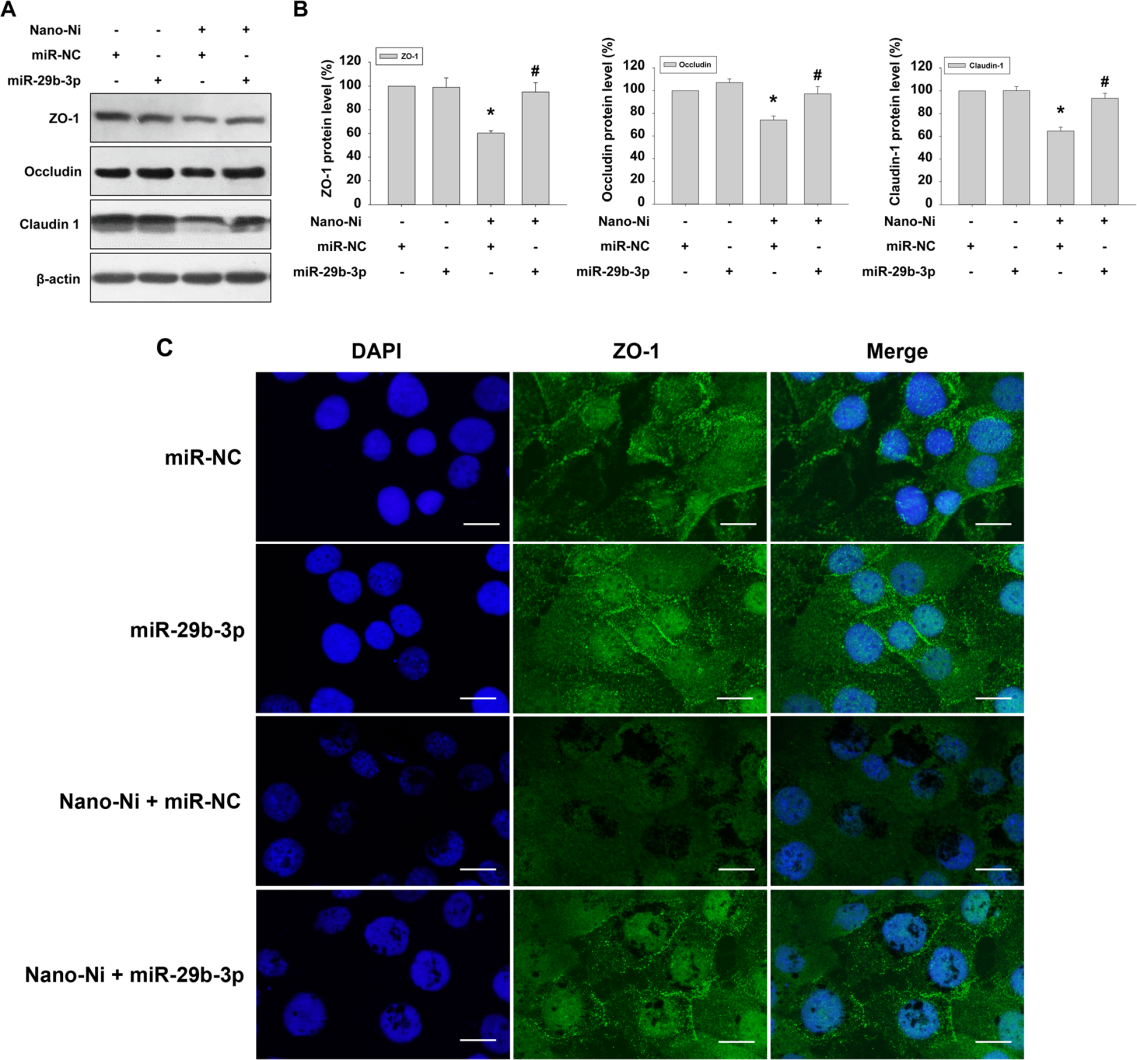
Effects of miR-29b-3p mimic on the expression of tight junction-associated proteins. Expression of tight junction-associated proteins in HaCaT cells were determined by Western blot and immunofluorescent staining after cells were transfected with miR-29b-3p mimic for 24 h prior to exposure to 20 μg/mL of Nano-Ni for another 24 h. A microRNA mimic negative control (miR-NC) was used to see if there are any off-targeting effects of miR-29b-3p mimic. **a** is the results of one experiment of Western blot, while **b** is normalized band densitometry readings averaged from three independent experiments ± SE. * Significant difference as compared with the control group, *p* < 0.05; # Significant difference as compared with the group with Nano-Ni treatment and negative control miR-NC transfection, *p* < 0.05. **c** presents the immunofluorescent staining of ZO-1 (green staining) in HaCaT cells. DAPI (blue) stains the whole nucleus of a cell. Scale bar represents 20 μm for all panels

To explore the effect of miR-29b on tight junction-associated proteins in HaCaT cells exposed to Nano-Ni, cells were transfected with miR-29b-3p mimic for 24 h followed by 20 μg/mL of Nano-Ni treatment for another 24 h. Transfection of miR-29b-3p mimic or miRNA negative control alone into cells did not cause any alteration of tight junction-associated proteins. However, miR-29b-3p mimic transfection significantly prevented cells from Nano-Ni-induced down-regulation of tight junction-associated proteins (Fig. 11a & b). The results of immunofluorescent staining also showed that miR-29b-3p mimic transfection restored Nano-Ni-induced reduced ZO-1 expression in HaCaT cells (Fig. 11c & Additional file 2).

## Discussion

The skin is the largest interface and barrier between the human body and the external environment. It plays an important role in controlling the entry and exit of substances, including water, electrolytes, biomolecules, and toxic substances [69]. With the development of nanotechnology and the wide application of nanomaterials, intentional or unintentional skin exposure to nanoparticles will inevitably increase and pose safety concerns. Previous studies have shown that metal nanoparticles can cross skin cells and hair follicles and exert toxic effects such as oxidative stress, DNA damage, and inhibition of mitochondrial activity [70, 71]. A previous study showed that exposure to Nano-Ni caused toxic effects on skin keratinocytes [72]. However, few studies have focused on the mechanisms that underlie these toxic effects. In this study, we first determined the alteration of expression and activities of MMP-2 and MMP-9 and the tight junction-associated proteins in HaCaT cells exposed to metal nanoparticles such as Nano-Ni. Then, we examined whether Nano-Ni would cause HIF-1α nuclear accumulation and dysregulation of miR-29b in HaCaT cells. Finally, we explored the possible mechanisms involved in the dysregulation of tight junction-associated proteins in HaCaT cells after exposure to Nano-Ni.

It is known that the pathogenesis of contact dermatitis is very complex. The mechanisms underlying the clinical symptoms vary depending on the allergic or non-allergic nature of dermatitis [73]. In the present study, HaCaT keratinocytes were selected because they are responsive to nickel exposure and are a useful model to assess metal cytotoxicity [74]. Previous studies have validated HaCaT cells as an in vitro model of allergic contact dermatitis, in which MMPs were elevated in response [75–77].

The present study revealed a dose-response cytotoxic effect on HaCaT cells after cells were exposed to various doses of Nano-Ni ranging from 0 to 50 μg/mL. The results of cell viability assays indicated that exposure to ≥30 μg/mL of Nano-Ni caused remarkable cytotoxic effects. However, exposure to the same doses of Nano-TiO_2_ did not significantly change the viability of HaCaT cells. Although our in vitro experiments cannot completely simulate actual Nano-Ni exposure in the human body or the quantity of Nano-Ni exposure that may be comparable to these dose-response studies, exposure to a dose that is lower than a cytotoxic dose can help identify potential health effects of Nano-Ni other than those due to cytotoxicity. Thus, non-cytotoxic doses of 10 μg/mL and 20 μg/mL were chosen to explore the other potentially toxic effects of Nano-Ni on human epidermal keratinocytes HaCaT and the potential mechanisms involved in these effects in our subsequent experiments.

Currently, only few occupational exposure limits exist specifically for nanomaterials. For example, the Occupational Safety and Health Administration (OSHA) recommends that worker exposure to nanoscale particles of TiO_2_ not exceed 0.3 mg per cubic meter (mg/m^3^), based on the National Institute for Occupational Safety and Health (NIOSH) proposed Recommended Exposure Limit (REL) (https://www.osha.gov/Publications/OSHA_FS-3634.html). Although TiO_2_ nanoparticles have been identified as an occupational workplace hazard by the NIOSH, exposure limits for nickel nanoparticles remain undefined. The existing exposure limits for nickel metal and ionic nickel are difficult to translate to nickel nanoparticle exposure; ionic nickel is soluble in physiological fluids and cell culture buffers, and exposure would result in different physiological effects than exposure to solid (non-soluble) nickel nanoparticles. According to OSHA occupational exposure levels (OELs), the workplace exposure limit for nickel metal is 0.5 mg/m^3^, assuming a worker is exposed for 8 h per workday, and metal nanoparticles could accumulate in the skin over time. However, daily occupational exposures likely vary, as it is extremely difficult to predict an accurate exposure given the variability in dust generation and ventilation among worksites. Therefore, it is difficult to estimate what a relevant nickel nanoparticle dosage or concentration should be for experimental models. In addition, accidental exposure to metal nanoparticles at the workplace cannot be ignored. There is a report about accidental exposure to nickel nanoparticles [15]. In that report, a worker was accidentally exposed to ~ 1 g of nickel nanoparticles during his 90 min of operating the process. The particulate nickel concentration in the vicinity of the operator was 382 mg/m^3^, and most of the particles were determined to be about 50 nm in diameter. The worker died from acute respiratory distress syndrome (ARDS). With the points described above in consideration, the doses we have chosen in this study are quite reasonable. Of note, although some metal nanoparticles, such as Nano-TiO_2_ or Nano-ZnO have been used in many commercial products such as cosmetics, no specific regulations exist; the current regulations are like those of other FDA regulated products that are not nanoparticles. Therefore, potential toxicity related to nanoparticle use warrants investigation.

Matrix metalloproteinases (MMPs) are a family of zinc-dependent endopeptidases that can be produced by a number of different types of cells in the skin, including fibroblasts, keratinocytes, macrophages, endothelial cells, mast cells, and eosinophils [78]. MMP-2 and MMP-9 are important members of the MMP superfamily, since they can degrade gelatin, collagen IV, V, VII and X, fibronectin, and elastin [79]. It is well known that MMPs play an important role in the proteolytic remodeling of extracellular matrix (ECM) in various physiologic situations, including developmental tissue morphogenesis, tissue repair, and angiogenesis. However, under pathologic conditions, overexpression of MMPs or insufficient control by tissue inhibitors of MMPs (TIMPs) results in the dysregulation of tissue remodeling and causes a variety of diseases such as cardiovascular diseases, pulmonary diseases, encephalomyelitis, rheumatoid arthritis, skin diseases, Alzheimer’s disease, and tumors [80]. A previous study showed that acute and chronic wounds were associated with high levels of MMP-2 and MMP-9, suggesting that non-healing ulcers developed an environment containing high levels of activated MMPs, which results in chronic tissue turnover and failure of wound closure [81]. MMP-9 has been implicated in blistering skin diseases and contact hypersensitivity [82, 83]. Exposure to nickel sulfate upregulated both MMP-2 and IL-8. However, increased expression of MMP-2 was found as early as 6 h after nickel sulfate exposure, while IL-8 upregulation occurred at 3 and 6 days after exposure [76]. Their findings suggested that MMPs could be used as sensitive indicators to screen the potential toxicity of new chemicals which could induce allergic contact dermatitis or other diseases whose pathogenesis involves active extracellular matrix degradation and remodeling.

In the present study, we found that Nano-Ni caused a dose- and time-dependent increase in the MMP-2 and MMP-9 expression and activities. Exposure of HaCaT to 20 μg/mL of Nano-Ni for as early as 12 h caused increased mRNA expression and/or activities of MMP-2 and MMP-9 but did not cause increased expression of IL-1β, IL-6, IL-18, and TNFα (data not shown). Upregulation of IL-6 and IL-18 was observed after HaCaT cells were exposed to 20 μg/mL of Nano-Ni for 24, 48, and 72 h (Data not shown). Our results were consistent with a previous study [84], where IL-18 production was observed in human keratinocytes exposed to dinitrochlorobenzene (DNCB) and paraphenylenediamine (PPD). These results suggest that MMPs are early responders following Nano-Ni exposure.

It is well known that activation of MMPs is a complex process. Regulation of MMPs may occur at multiple levels, either by gene transcription and synthesis of inactive pro-enzymes, post-translational activation of pro-enzymes, or via the interaction of secreted MMPs with their inhibitors called TIMPs [85, 86]. TIMPs are a group of smaller (20–30 kDa) MMP inhibitors, and four TIMPs (TIMP-1 to TIMP-4) have been identified and characterized [87]. Strong expression and synthesis of TIMP-1 and TIMP-2, as well as upregulation of MMP-2 and membrane type 1 MMP (MT1-MMP), have been found in bone marrow-derived human mesenchymal stem cells (hMSCs) [88]. Our previous studies also found that Nano-Ni caused upregulation of MMP-2 and MMP-9, as well as TIMP-1 and/or TIMP-2 in primary mouse peripheral blood monocytes or human monocytes U937 [50, 59].

The role of TIMPs in physiological and pathological conditions is complex. Except as endogenous inhibitors of MMPs, TIMPs serve other functions as well. For example, TIMP-2 acts as a co-activator in pro-MMP-2 activation, which is thought to take place on the cell surface. TIMP-2 binds to membrane-tethered membrane type 1 MMP (MT1-MMP or MMP-14) and pro-MMP-2 with a 1:1 stoichiometry to form a ternary complex, thus recruiting secreted pro-MMP-2 on the cell surface and leading to the activation of pro-MMP-2 by another free MT1-MMP [89–92]. Although no obvious defect in TIMP-2-deficient mice was found, lack of TIMP-2 altered the processing of pro-MMP-2, suggesting the requirement of TIMP-2 for efficient activation of pro-MMP-2 both in vivo and in vitro [89, 90, 92]. The hemopexin domain of pro-MMP-9 also forms a tight complex with TIMP-1 and TIMP-3 through their C-terminal domains [93]. The role of the pro-MMP-9/TIMP-1 or 3 complex is still unknown. Previous studies demonstrated that independent of MMP-9 inhibition, TIMP-1 acts as signaling molecules with cytokine-like activities thereby influencing various biological processes including cell growth, apoptosis, differentiation, angiogenesis, and oncogenesis [94]. Therefore, more specific biochemical studies at the molecular level may be needed to explore the effects of Nano-Ni on the balance between MMPs and TIMPs in HaCaT cells.

Both MMP-2 and MMP-9 participate in the progress of various diseases by degrading tight junction-associated proteins in the blood-brain barrier, blood-testis barrier, skin barrier, and so on [95]. A previous study showed that MMP-9 knockout attenuated the blood-brain barrier disruption by reducing the degradation of ZO-1 protein in mice [96]. MMP-2 and MMP-9 derived from leukemic cells upregulated the permeability of the blood-brain barrier by disrupting tight junction-associated proteins [97]. In addition, increased MMP-9 expression/activity disrupted blood-testis barrier via down-regulation of occludin and connexin 43 [35]. MMP-9 also participated in asthma-related epithelial structural remodeling and functional damage by changing the immune localization of tight junction [98]. In this study, we found that Nano-Ni exposure induced increased expression and activities of MMP-2 and MMP-9 in HaCaT cells; therefore, it is important to investigate whether exposure to metal nanoparticles could cause dysregulation of tight junction-associated proteins. We determined the expression of tight junction-associated proteins in HaCaT cells with Nano-Ni exposure by Western blot. Our data revealed a dose- and a time-dependent decrease in the expression of claudin-1, occludin, and ZO-1 in HaCaT cells exposed to Nano-Ni. Tight junction-associated proteins have been indicated as matrix metalloproteinase substrates [99], thus our results suggested that Nano-Ni-induced activation of MMP-2 and MMP-9 and the imbalance of the MMP/TIMP system may be involved in the Nano-Ni-induced down-regulation of tight junction-associated proteins. However, further studies are needed to explore how Nano-Ni-induced activated MMPs regulate tight junction-associated proteins.

HIF-1 is a heterodimer consisting of an unstable alpha subunit (HIF-1α) and a stable beta subunit (HIF-1β) [100]. HIF-1α is rapidly degraded under normoxic conditions, but translocates from the cytoplasm to the nucleus when oxygen supply is limited and forms a stable heterodimer with HIF-1β [100]. HIF-1 signaling can cause up-regulation of genes that are involved in angiogenesis, glucose metabolism, cell proliferation/survival, and invasion/metastasis [101]. Nickel is known to mimic hypoxia through the activation of HIF-1α [101–104]. Treatment with nickel leads to a rapid and concentration-dependent stabilization and nuclear accumulation of HIF-1α [105]. Upon dimerization of HIF-1α with another subunit of HIF, HIF-1β, HIF-1 binds to hypoxia response elements (HRE) in target genes, such as VEGF and MMPs and activates them [101, 102]. Our previous studies demonstrated that exposure of U937 or A549 cells to Nano-Ni caused HIF-1α nuclear accumulation [50, 106]. In this study, we found that Nano-Ni exposure caused HIF-1α nuclear accumulation in human epidermal keratinocytes HaCaT. The exact mechanism about how nickel induces HIF-1α nuclear accumulation is still unclear. Previous studies have shown that nickel can deplete intracellular ascorbate, resulting in inactivation of prolyl hydroxylases (PHDs) and hypoxia-like stress [101, 103, 107]. Another possibility is that nickel can substitute for Fe^2+^ in the regulatory dioxygenases including PHDs and this substitution inactivates the enzymes. Nickel may also bind more tightly than Fe^2+^ to the membrane transporter DMT-1 and suppress the delivery of Fe^2+^ into cells, causing depletion of intracellular Fe^2+^ [101, 108]. Other components in the canonical mechanism of HIF-1α stabilization can also be affected by nickel. In addition, HIF-1α also can be regulated by PHD-independent mechanisms, for example, mitochondrial respiratory complexes damage may also stabilize HIF-1α [109]. The possibility that nickel may affect molecules involved in other HIF-1α stabilization mechanisms cannot be completely excluded.

Although TLR2 and TLR4 signaling may play important roles in allergic contact dermatitis [84, 110], there is no unifying mechanism underlying contact allergen-induced allergic contact dermatitis, and each allergen may exert differential cellular effects based on its specific physicochemical and immunological properties and corresponding interactions with biomolecules. A previous study has demonstrated that HIF-1α acts as an important pathway involved in contact hypersensitivity reactions to nickel, and the HIF-1α pathway and IKK2/NF-κB pathway act independently to regulate expression of nonoverlapping gene pools [105]. NF-κB activation mediates most of the proinflammatory responses to nickel, while Nickel-dependent HIF-1α activation primarily modulates the expression of genes involved in proliferation, survival, metabolism, and signaling [105]. In this study, exposure to Nano-Ni rapidly caused nuclear accumulation of HIF-1α. Our results also showed that Nano-Ni-induced HIF-1α nuclear accumulation was prevented after cells were pre-treated with a HIF-1α inhibitor, CAY10585, which has been proven to promote proteasomal degradation of HIF-1α via upregulation of von Hippel-Lindau (VHL) without affecting HIF-1β expression [65, 66]. Thus, we also used CAY10585 to pretreat HaCaT cells to determine whether Nano-Ni-induced HIF-1α nuclear accumulation was involved in the Nano-Ni-induced MMP upregulation and miR-29b down-regulation.

Hypoxia and ischemia could change the expression profiles of many miRNAs [111]. A limited number of these so-called hypoxamiRs has been identified, which can be affected by HIF expression [112, 113]. For example, hypoxic stress down-regulated miR-29b in cardiomyocytes [62, 64]. Oxygen-glucose deprivation reduced miR-29b expression in microglia [114]. The expression level of miR-29b decreased in ischemia/reperfusion (I/R) -induced cells and I/R-induced intestinal tissues of mice as compared with control or sham group [63]. In addition, hypoxia reduced the expression of miR-29b by 40% after 1 day and 60% after 5 days in rat cerebral cortex [115]. In this study, we found that Nano-Ni caused down-regulation of miR-29b, which could be prevented when cells were pretreated with CAY10585, a HIF-1α inhibitor, suggesting Nano-Ni-induced HIF-1α nuclear accumulation is involved in Nano-Ni-induced down-regulation of miR-29b. Although other factors including reactive oxygen species (ROS) may also decrease miR-29b expression, our results showed that both Nano-Ni and Nano-TiO_2_ did not cause any significant ROS generation in HaCaT cells (data not shown) and excluded the potential role of ROS on the decreased miR-29b expression.

miRNAs are endogenous small non-coding RNAs with the capacity to regulate gene expression post-transcriptionally. miRNA mainly binds to the complementary seed sequence in the untranslated region (3′-UTR) of the target mRNA under the guidance of the RNA-induced silencing complex, thereby promoting mRNA degradation or inhibiting protein translation. The miR-29 family consists of miR-29a, miR-29b, and miR-29c, among which miR-29b is the most highly expressed and is found at two genomic loci [116]. Exposure to some metal nanoparticles has exhibited epigenetic effects, inducing altered expression of miRNA, in various toxicological models in vivo and in vitro [117]. A previous study showed that exposure of THP-1 cells to silver, titanium dioxide, and zinc oxide nanoparticles caused significant alteration of miRNA expression profile via small RNA sequencing, and down-regulation of miR-142-3p amplified or promoted the phagocytosis of nanoparticles [118]. Exposure to silica nanoparticles caused spermatocyte autophagy through miR-494 targeting AKT in GC-2spd cells [119]. In this study, we used prediction algorithms and found that miR-29b targets both MMP-2 and MMP-9 directly by binding to the complementary seed sequence (GGUGCU) in the untranslated region (3′-UTR) of MMP-2 and MMP-9 mRNA. In fact, previous studies have demonstrated that miR-29b-3p directly modulated the expression of MMP-2 and MMP-9 in the tumor microenvironment and thus indirectly regulate epithelial plasticity [45]. We found here that although low-dose (10 μg/mL) of Nano-Ni caused significant miR-29b downregulation, this dose only caused a slight, but not significant increase in MMP-2 and MMP-9 mRNA expression. Nano-Ni exposure may not deplete all endogenous miR-29b; the remaining miR-29b may bind MMP-2 and MMP-9 mRNA directly, resulting in their degradation and diminishing Nano-Ni-induced increased MMP-2/9 mRNA expression. In order to investigate whether Nano-Ni-induced down-regulation of miR-29b was involved in Nano-Ni-induced upregulation of MMPs and down-regulation of tight junction-associated proteins, miR-29b-3p mimic was used to transfect HaCaT cells. Our results demonstrated that miR-29b-3p mimic transfection inhibited Nano-Ni-induced increased expression and activities of MMP-2 and MMP-9 and restored Nano-Ni-induced down-regulation of tight junction-associated proteins such as claudin-1, occludin, and ZO-1 in HaCaT cells, suggesting the involvement of miR-29b in Nano-Ni-induced activation of MMP-2 and MMP-9 and down-regulation of tight junction-associated proteins.

In this study, Nano-TiO_2_ was used as a control. Although Ti and Ni are both transition metals, different metals may pose different cytotoxic, immunological, and genotoxic effects due to their different physical and chemical properties. Nano-TiO_2_ has been widely used in industry, especially in commercially available sunscreens. Many critical points in the current literature contribute to the controversy surrounding the toxic, immunological effects, or genotoxic effects of Nano-TiO_2_. The effects depend on the type and amount of Nano-TiO_2_ used, exposure time, and exposure route. Our previous studies have shown that exposure to Nano-TiO_2_ did not cause HIF-1α nuclear accumulation and MMP-2/9 upregulation in human monocytes [50, 51]. Previous studies showed that the uptake of Nano-TiO_2_ into the cultured cells was restricted to phagosomes; Nano-TiO_2_ did not enter the nucleus or any other cytoplasmic organelle. No other morphological changes were detected after 24 h exposure [120]. Topical exposure to TiO_2_ nanoparticles did not induce skin sensitization; however, subcutaneous injection of TiO_2_ nanoparticles resulted in significant increases in lymphocyte proliferation, suggesting that the inability of TiO_2_ nanoparticles to penetrate the skin might be a limiting factor in the potential to induce skin sensitization [52, 53]. The European Union (EU)‘s Scientific Committee on Consumer Safety (SCCS) approved nanometric titanium dioxide (in the three crystalline forms) to be considered safe for use in cosmetic products intended for application on healthy, intact, or sunburnt skin [121]. In this study, we found that Nano-Ni, but not Nano-TiO_2_, exposure caused increased mRNA expression and activities of MMP-2 and MMP-9 and decreased tight junction-associated proteins, suggesting Nano-Ni, but not Nano-TiO_2_, may penetrate the skin to induce skin sensitization by disrupting the skin barrier.

## Conclusions

**Fig. 12:**
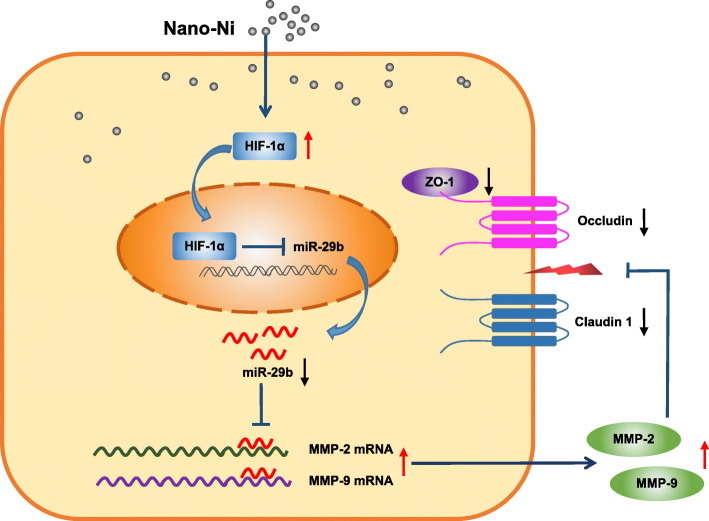
Schematic and potential mechanisms of Nano-Ni-induced dysregulation of tight junction-associated proteins in human epidermal keratinocytes. Exposure of human epidermal keratinocytes to Nano-Ni caused HIF-1α nuclear accumulation, down-regulation of miR-29b, and upregulation of MMP-2 and MMP-9, and finally resulted in dysregulation of tight junction-associated proteins. Our results demonstrate that Nano-Ni-induced dysregulation of tight junction-associated proteins in skin keratinocytes is via HIF-1α/miR-29b/MMPs pathway

Taken together, our study herein demonstrated that exposure of human epidermal keratinocytes to Nano-Ni caused increased transcription and activities of MMP-2 and MMP-9 and dysregulation of tight junction-associated proteins. Our results also revealed that Nano-Ni-induced miR-29b down-regulation was through HIF-1α nuclear accumulation caused by Nano-Ni. HaCaT cells transfected with miR-29b-3p mimic prior to exposure to Nano-Ni restored Nano-Ni-induced activation of MMP-2 and MMP-9 and dysregulation of tight junction-associated proteins. All the above results demonstrate that Nano-Ni-induced dysregulation of tight junction-associated proteins in skin keratinocytes is via the HIF-1α/miR-29b/MMPs pathway (Fig. 12). The results provide a further understanding of dermatologic toxicity caused by Nano-Ni exposure. However, the precise mechanisms underlying Nano-Ni-induced skin sensitivity and injury need to be further investigated. Nano-Ni-induced inflammation and cytokine production and their associated signaling pathways may also be involved in Nano-Ni-induced dermatologic toxicity, which cannot be ignored. Our in vitro results need to be further augmented by studies of Nano-Ni-induced allergic contact hypersensitivity in vivo. In addition, exposure levels of Nano-Ni in the working environment should be determined and epidemiological studies regarding contact dermatitis in worker exposure to Nano-Ni need to be investigated.

## Supplementary Information

**Additional file 1. **Quantification of ZO-1 expression in Fig. 5e**.** ZO-1 expression (green staining) in HaCaT cells exposed to 20 μg/mL of Nano-Ni or Nano-TiO_2_ for 24 h was determined by immunofluorescent staining, and quantified by NIH ImageJ software (http://imagej.nih.gov/ij/). The cells without any treatments were used as control. Values are mean ± SE of three independent experiments. * Significant difference as compared with the control group, *p* < 0.05.

**Additional file 2. **Quantification of ZO-1 expression in Fig. 11c**.** ZO-1 Expression in HaCaT cells were determined by immunofluorescent staining after cells were transfected with miR-29b-3p mimic for 24 h prior to exposure to 20 μg/mL of Nano-Ni for another 24 h. A microRNA mimic negative control (miR-NC) was used to see if there are any off-targeting effects of miR-29b-3p mimic. ZO-1 expression was quantified by NIH ImageJ software (http://imagej.nih.gov/ij/). Values are mean ± SE of three independent experiments. * Significant difference as compared with the control group, *p* < 0.05; # Significant difference as compared with the group with Nano-Ni treatment and negative control miR-NC transfection, *p* < 0.05.

## Data Availability

All data and materials are included in the manuscript.

## Abbreviations

Nano-Ni: Nickel nanoparticles
Nano-TiO_2_: Titanium dioxide nanoparticles
HIF-1α: Hypoxia inducible factor 1α
MMP-2: Matrix metalloproteinase-2
MMP-9: Matrix metalloproteinase-9
miR-29b: microRNA-29b
TIMP-1: Tissue inhibitor of metalloproteinases 1
TIMP-2: Tissue inhibitor of metalloproteinases 2
ZO-1: Zonula occludens-1
CAY10585: 4-hydroxy-3-[[2-(4-tricyclo[3.3.1.13,7]dec-1-ylphenoxy)acetyl]amino]-benzoic acid methyl ester
