# Supplementary figures and images for: Effects of metal nanoparticles on tight junction-associated proteins via HIF-1α/miR-29b/MMPs pathway in human epidermal keratinocytes
Source: Part Fibre Toxicol. 2021 Mar 19;18:13. doi: 10.1186/s12989-021-00405-2 (PMC7980342; doi:10.1186/s12989-021-00405-2)

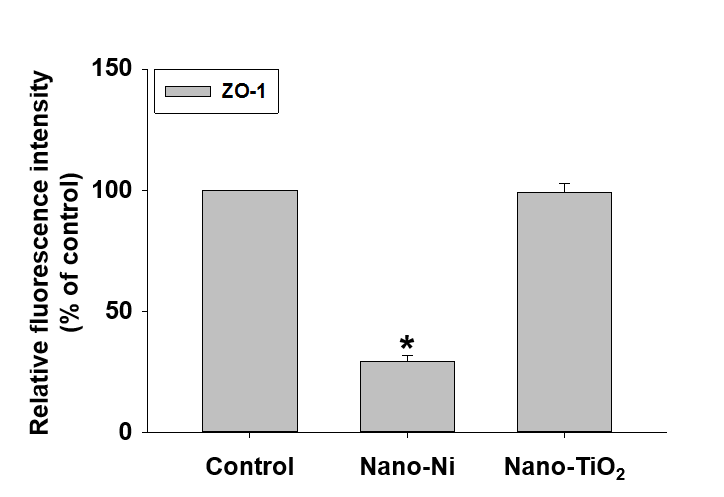

Supplement: Supplementary file 1 — Additional file 1. Quantification of ZO-1 expression in Fig. 5e. ZO-1 expression (green staining) in HaCaT cells exposed to 20 μg/mL of Nano-Ni or Nano-TiO2 for 24 h was determined by immunofluorescent staining, and quantified by NIH ImageJ software (http://imagej.nih.gov/ij/). The cells without any treatments were used as control. Values are mean ± SE of three independent experiments. * Significant difference as compared with the control group, p < 0.05. [file 12989_2021_405_MOESM1_ESM.tif]

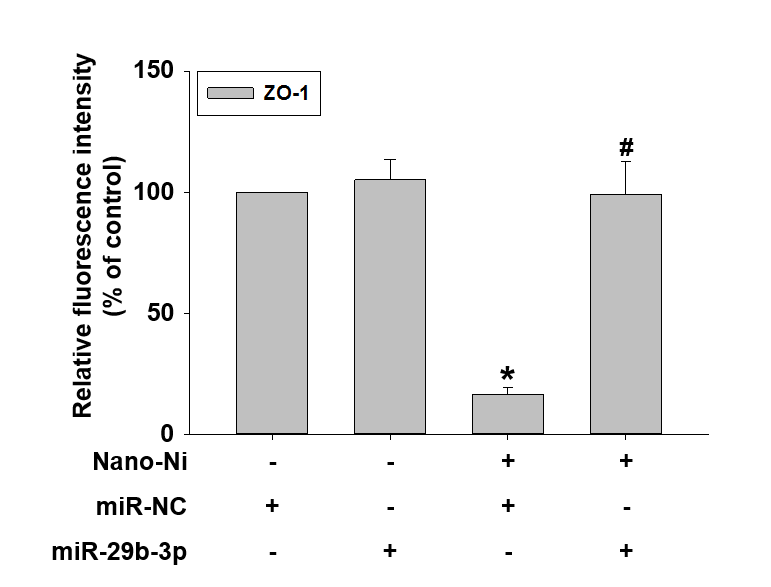

Supplement: Supplementary file 2 — Additional file 2. Quantification of ZO-1 expression in Fig. 11c. ZO-1 Expression in HaCaT cells were determined by immunofluorescent staining after cells were transfected with miR-29b-3p mimic for 24 h prior to exposure to 20 μg/mL of Nano-Ni for another 24 h. A microRNA mimic negative control (miR-NC) was used to see if there are any off-targeting effects of miR-29b-3p mimic. ZO-1 expression was quantified by NIH ImageJ software (http://imagej.nih.gov/ij/). Values are mean ± SE of three independent experiments. * Significant difference as compared with the control group, p < 0.05; # Significant difference as compared with the group with Nano-Ni treatment and negative control miR-NC transfection, p < 0.05. [file 12989_2021_405_MOESM2_ESM.tif]
